# The Nucleolar Protein Nucleophosmin Is Physiologically Secreted by Endothelial Cells in Response to Stress Exerting Proangiogenic Activity Both In Vitro and In Vivo

**DOI:** 10.3390/ijms22073672

**Published:** 2021-04-01

**Authors:** Anna Di Carlo, Sara Beji, Silvia Palmerio, Mario Picozza, Marco D’Agostino, Vincenzo Petrozza, Roberta Melchionna, Antonia Germani, Alessandra Magenta, Elena De Falco, Daniele Avitabile

**Affiliations:** 1 Tumor Immunology and Immunotherapy Unit, IRCCS Regina Elena National Cancer Institute, 00144 Rome, Italy; anna.dicarlo@ifo.gov.it (A.D.C.); melchionna@ifo.gov.it (R.M.); 2 Laboratory of Experimental Immunology, Istituto Dermopatico dell’Immacolata, IDI-IRCCS, 00167 Rome, Italy; bejisara@gmail.com (S.B.); palmerio.sil@gmail.com (S.P.); marcodagostino86@hotmail.it (M.D.); a.germani@idi.it (A.G.); 3 Neuroimmunology Unit, IRCSS Fondazione Santa Lucia, 00143 Rome, Italy; m.picozza@hsantalucia.it; 4 Department of Medical Surgical Sciences and Biotechnologies, Sapienza University, 04100 Latina, Italy; vincenzo.petrozza@uniroma1.it (V.P.); elena.defalco@uniroma1.it (E.D.F.); 5 Institute of Translational Pharmacology (IFT), Consiglio Nazionale delle Ricerche (CNR), 00133 Rome, Italy; ale.magenta@gmail.com; 6 Mediterranea Cardiocentro, 80122 Naples, Italy; 7 Department of Scientifico e Sviluppo, IDI Farmaceutici, Via dei Castelli Romani 73/75, 00071 Pomezia, Italy

**Keywords:** nucleophosmin, HUVEC, angiogenesis, *VEGF*, inflammation, alarmin

## Abstract

Nucleophosmin (NPM), a nucleolar multifunctional phosphoprotein, acts as a stress sensor in different cell types. NPM can be actively secreted by inflammatory cells, however its biology on endothelium remains unexplored. In this study, we show for the first time that NPM is secreted by human vein endothelial cells (HUVEC) in the early response to serum deprivation and that NPM acts as a pro-inflammatory and angiogenic molecule both in vitro and in vivo. Accordingly, 24 h of serum starvation condition induced NPM relocalization from the nucleus to cytoplasm. Interestingly, NPM was increasingly excreted in HUVEC-derived conditioned media in a time dependent fashion upon stress conditions up to 24 h. The secretion of NPM was unrelated to cell necrosis within 24 h. The treatment with exogenous and recombinant NPM (rNPM) enhanced migration as well as the Intercellular Adhesion Molecule 1 (ICAM-1) but not Vascular cell adhesion protein 1 (VCAM-1) expression and it did not affect cell proliferation. Notably, in vitro tube formation by Matrigel assay was significantly increased in HUVEC treated with rNPM compared to controls. This result was confirmed by the in vivo injection of Matrigel plug assay upon stimulation with rNPM, displaying significant enhanced number of functional capillaries in the plugs. The stimulation with rNPM in HUVEC was also associated to the increased expression of master genes regulating angiogenesis and migration, including Vascular Endothelial Growth Factor-A (*VEGF-A*), Hepatocyte Growth Factor (*HGF*), Stromal derived factor-1 (*SDF-1*), Fibroblast growth factor-2 (*FGF-2*), Platelet Derived Growth Factor-B (*PDGF-B*), and Matrix metallopeptidase 9 (*MMP9*). Our study demonstrates for the first time that NPM is physiologically secreted by somatic cells under stress condition and in the absence of cell necrosis. The analysis of the biological effects induced by NPM mainly related to a pro-angiogenic and inflammatory activity might suggest an important autocrine/paracrine role for NPM in the regulation of both phenomena.

## 1. Introduction

Alarmins are endogenous molecules released upon tissue damage and able to modulate the response to stress by actively interacting with the immune system [1]. On the other hand, the uncontrolled and excessive release of alarmins contributes to deregulate several processes, which can progress towards chronic inflammatory and autoimmune phenomena. According to their dual function role, alarmins are biologically important because they also orchestrate tissue repair and angiogenesis when the endothelium is not injured and under multiple physiological stress conditions not necessarily correlated with necrosis or apoptosis [2,3]. 

Recently, increasing evidence is growing on the specific alarmin nucleophosmin (NPM), an endogenous and abundant nucleolar multifunctional protein, belonging to a histone chaperones family, with shuttling properties from the nucleus to the cytoplasm, and a main contributor to a wide range of metabolic processes including ribosome biogenesis, proliferation, migration, cell cycle control, DNA repair, non-programmed cell death, and sensor of stress [4,5]. 

So far, the role of NPM in disease development has been extensively explored mainly in cancer, given its main involvement in genomic stability and in cell transformation [6,7], where NPM has been demonstrated to act both as oncogene and oncosuppressor in leukemias as well as in solid tumors [8,9,10,11]. 

In addition to carcinogenesis, NPM has been also implicated in the activation and maintenance of inflammation and vascular diseases such as endothelial oxidative stress-related disorders, aging, atherosclerosis [12,13], hypertension, and cardiovascular diseases [14]. Moreover, NPM has been found to be actively secreted by vascular inflammatory cells [15] and by endotoxin stimulated macrophages [16]. The knockdown of NPM in endothelial cells inhibits inflammatory soluble mediators and adhesion molecules such as Intercellular Adhesion Molecule 1 (ICAM-1) and Vascular cell adhesion protein 1 (VCAM-1) [15]. These studies clearly suggest the contribution of NPM in the pathophysiology of endothelium and in the maintenance of endothelial homeostasis and counteraction of the exogenous stress.

Nevertheless, the contribution of NPM under physiological conditions remains unexplored as well as the capacity of the non-injured endothelium to potentially respond to NPM. Accordingly, the well acknowledged alarmins such as the High mobility group box 1 protein (HMGB-1) showing similar properties to NPM, have been recognized to regulate angiogenesis [17]. Thus, analogous angiogenic functions could be attributed to NPM.

In this study we investigated the extracellular biological functions of NPM and the in vivo angiogenic role in human vein endothelial cells (HUVEC), an acknowledged model for macrovascular endothelium. In fact, insights on the secretive properties of NPM with specific regard to the active role of NPM in the endothelial cellular response to stress, still requires to be properly addressed.

We show for the first time that NPM is secreted by HUVEC under stress condition but in absence of cell necrosis and that NPM is able to enhance migration, exhibiting gene expression profile associated to pro-inflammation and angiogenic activity associated to angiogenesis both in vitro and in vivo.

## 2. Results

**Figure 1:**
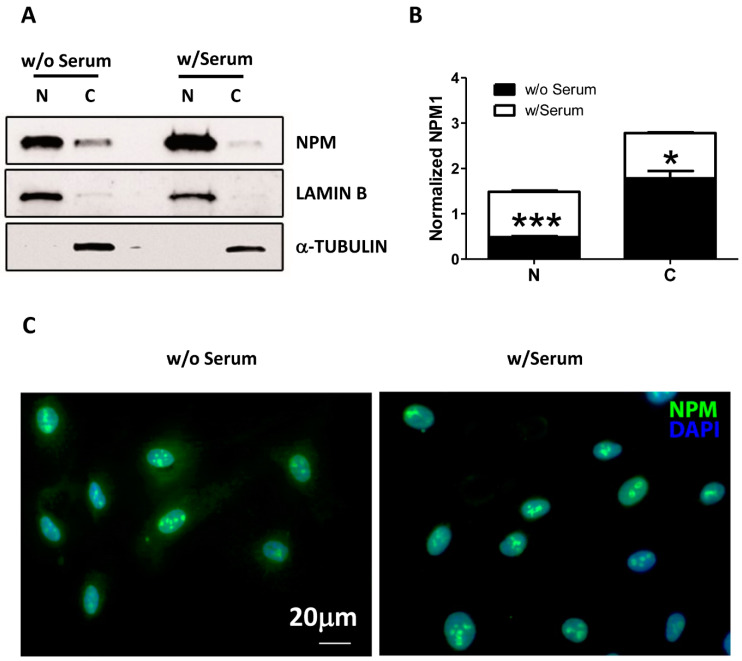
(**A–C**)**.** Western Blotting analysis of cytoplasm and nuclear fraction of nucleophosmin (NPM) in human vein endothelial cells (HUVEC) after serum starvation. (**A**) Representative blot displaying the shuttle of NPM. Lamin B and α-tubulin have been employed as nuclear and cytoplasmic protein, respectively. (**B**) The densitometry analysis shows that NPM translocates from nucleus to the cytoplasm after serum deprivation (w/o Serum) but not in the presence of serum (w/Serum). Data are the results of 3 independent experiments. * *p* < 0.05, *** *p* < 0.001. (**C**) Immunofluorescent staining of HUVEC confirming a delocalized NPM in the cytoplasm in the absence of serum (w/o Serum) but not in presence of serum (w/Serum). NPM and DAPI stain green and blue, respectively. Scale bar is displayed.

Given the ability of NPM to act as stress sensor protein by delocalizing from the nucleolus to the cytoplasm under stress condition [10,11], we first examined whether serum and growth factor deprivation could induce a similar phenomenon in endothelial cells (HUVEC) by performing a cell fractionation assay. In complete medium cultured cells, Western blot analysis confirmed the expected pattern for NPM distribution, which was predominantly detected in the nucleus and slightly in the cytoplasm compartment of HUVEC (Figure 1A,B). However, upon 24 h of serum deprivation, we observed a significant accumulation of NPM in cytoplasm of HUVEC and a concomitant decrease in the nucleus compartment (Figure 1A,B). The result was confirmed by immunofluorescence staining mirroring a similar scenario after 24 h (Figure 1C).

**Figure 2:**
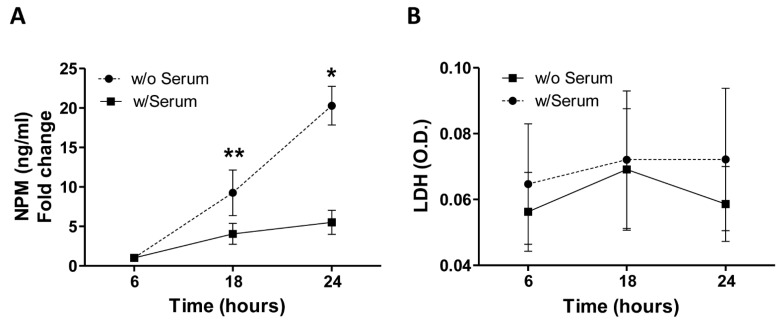
(**A**,**B**). Secretion of NPM by HUVEC in serum starvation conditions and in absence of cell necrosis. (**A**) Active release of NPM into the microenvironment of HUVEC according to a time course (6, 18, 24 h) of serum deprivation (w/Serum) and measured by ELISA, showing a gradual increase of the protein in the culture media of endothelial cells at 18 and 24 h compared to 6 h. Data are the results of 4 independent experiments (technical duplicates). * *p* < 0.05, ** *p* < 0.01. (**B**) Concentration of the enzyme lactate dehydrogenase (LDH) displaying the absence of cell necrosis in both conditions (with Serum, w/Serum and without Serum, w/o Serum). Data are the results of 3 independent experiments (technical duplicates). O.D. optical density.

Based on this observation, we further investigated whether the cytoplasmic translocation of NPM could also involve its release in the extracellular microenvironment. To this aim, after serum deprivation HUVEC-derived conditioned media were collected according to a defined time course (6, 18, and 24 h) and the amount of NPM was quantified by ELISA. We found that soluble levels of NPM progressively tended to increase over the time peaking at 24 h of stress condition (Figure 2A, *p* < 0.01 and *p* < 0.05, 18 and 24 h vs. 6 h, respectively). As the release of NPM by necrotic cells has been described [7], in order to rule out potential passively necrosis-related secretion of NPM, we also measured the release of the enzyme lactate dehydrogenase (LDH), a marker of membrane integrity. Importantly, no significant changes of LDH levels were detected in the supernatants of HUVEC, as cells expanded under serum deprivation and standard conditions with serum showed a comparable profile at all time points (Figure 2B, all *p* > 0.05).

**Figure 3:**
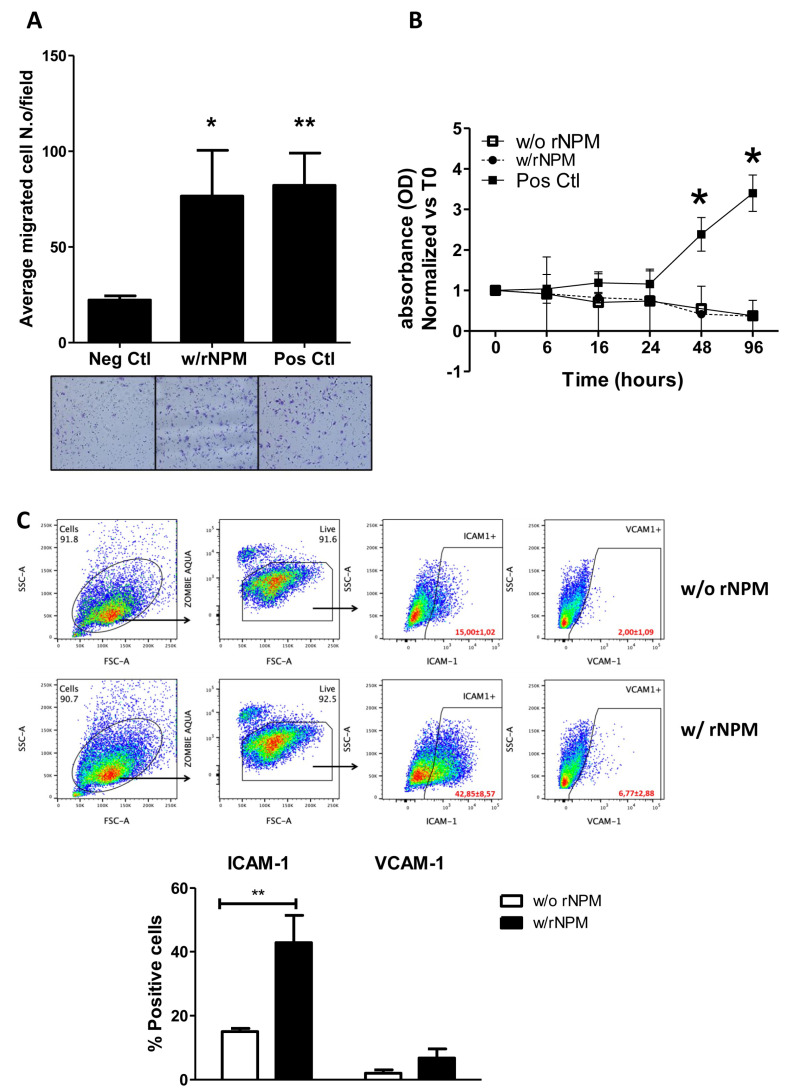
(**A**–**C**). Biological effects of exogenous NPM on endothelial cells. (**A**) The graph shows that the exogenous recombinant NPM (rNPM 200 ng/mL, w/rNPM) is able to foster migration of HUVEC. The 10% Fetal Bovine Serum (FBS) and the EBM^TM^-2 have been used as positive and negative stimulus, respectively (Pos Ctl, positive control; Neg Ctl, negative control). Below the graph are representative images of migrated cells. Magnification 4X. Data are the results of 5 independent experiments (technical duplicates). * *p* < 0.05, ** *p* < 0.01. (**B**) Proliferation assay (MTS assay) showing that the increased migration of HUVEC upon rNPM treatment is not associated to an enhancement of the proliferation up to 96 h. Data are the results of 4 independent experiments (technical duplicates). *p < 0.05. Complete medium has been used as the positive control (Pos Ctl). O.D., optical density, normalized vs. time 0. (**C**) FACS Analysis displaying scatter plots (top panel) with gating strategy to analyze only viable cells for ICAM-1 and VCAM-1 expression in the presence (w/rNPM) or in the absence of rNPM (w/o rNPM) treatment. Quantification plot (Bottom panel) showing that rNPM can significantly increase the percentage of positive HUVEC for ICAM-1 but not for VCAM-1. Data are the results of 4 independent experiments. ** *p* < 0.01.

Considering that the endogenous NPM was secreted by HUVEC, we aimed to explore its paracrine properties on endothelial cells. Specifically, we assessed both migrative and proliferative ability of HUVEC after stimulation with exogenous recombinant NPM (rNPM 200 ng/mL, which was assessed by a curve dose–response) and also suggested by the FACS Analysis showing 200 ng/mL rNPM as the best physiological concentration). Results showed that rNPM significantly increased the chemotactic property of HUVEC compared to the negative control (EBM-2^TM^), Figure 3A, *p* < 0.05). The enhancement of cell migration was not caused by a proliferative effect. In fact, the exogenous stimulation of HUVEC with rNPM did not induce cell proliferation up to 96 h compared to the negative control (Figure 3B, *p* > 0.05). Additionally, the potential modulation of inflammatory markers expressed on endothelial cells and induced by rNPM was investigated. To this aim, HUVEC were treated with rNPM (200 ng/mL) for 24 h and analyzed by FACS for the expression of CD54 (ICAM-1) and CD106 (VCAM-1), the constitutive and activated main markers involved in the inflammatory modulation [13]. Interestingly, the percentage of positive endothelial cells to ICAM-1 but not to VCAM-1 significantly increased after treatment with rNPM (Figure 3C, *p* < 0.01).

**Figure 4:**
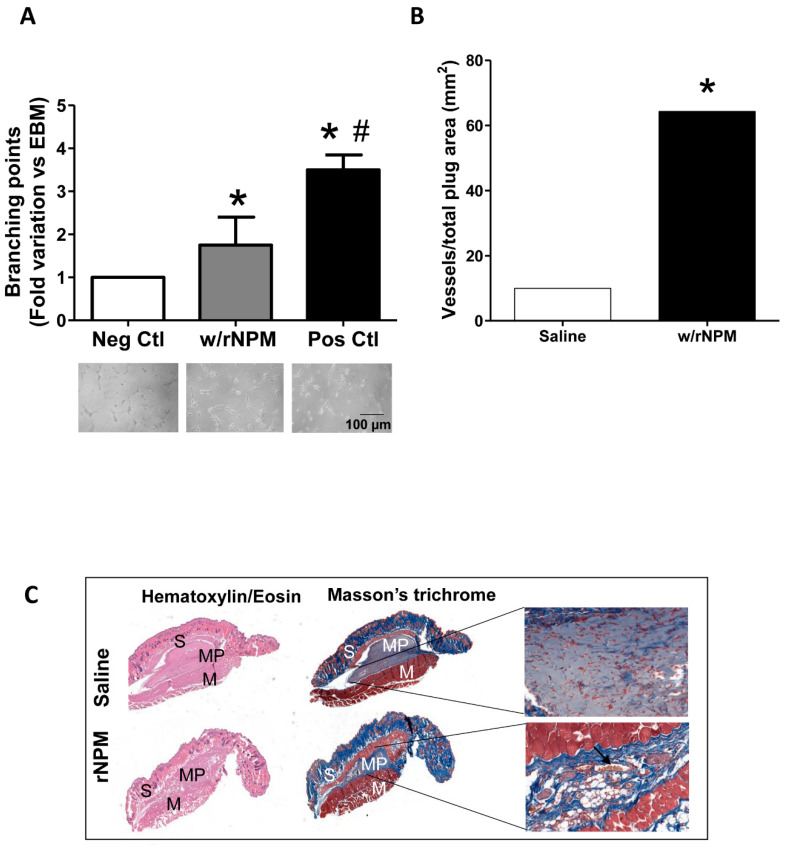
(**A**–**C**). In vitro and in vivo effects of rNPM on endothelial cells. (**A**) The graph shows that the treatment with rNPM significantly enhances the number of in vitro branching points in Matrigel assays respect to the negative control (Neg Ctl, EBM^TM^-2). The 10% FBS is the positive control (Pos Ctl). Data are the results of 3 independent experiments (technical duplicates). * *p* < 0.05, # *p* < 0.001. The representative optical images of the angiogenic assay in culture in the corresponding conditions of the graph are shown below the graph. Scale bar is displayed. (**B**) In in vivo Matrigel plugs, the number of vessels/area is significantly enhanced after treatment with rNPM respect to saline. Data are the results of 3 independent experiments. * *p* < 0.05. (**C**) Representative images of the whole section of the plugs stained for hematoxylin/eosin and Masson’s trichrome after treatment with saline or rNPM (w/rNPM) in the Matrigel plugs. Magnification 4×. S, skin, MP, Matrigel plug, M, muscle. The insert magnified (20×) displayed the presence of red blood cells, the hallmark of functional formed vessels in the plug. Erythrocytes within the capillary lumen are indicated by the arrow.

Next, to assess whether rNPM was able to modulate endothelial cell function by exerting potential angiogenic activity, we performed an in vitro Matrigel assay. The 10% FBS condition was used as the positive control [14]. Results showed that rNPM induced a significant increase of the number of branching points compared to the negative control (Figure 4A, *p* < 0.05). More importantly, this result was confirmed by in vivo experiments where Matrigel supplemented with 200 ng/mL rNPM was injected subcutaneously into the mid-lower abdominal region of mice, in order to foster the migration of host endothelial toward the formation of vascular networks in the Matrigel plugs. After 8 days the residual plugs were evaluated by H&E and Masson’s trichrome staining, by quantifying the newly formed functional vessels in the plug, identified by the presence of erythrocytes within [15,16]. Results confirmed a significant increase of the number of in vivo functional vessels after injection of rNPM compared to saline (Figure 4B,C, *p* < 0.001).

**Figure 5:**
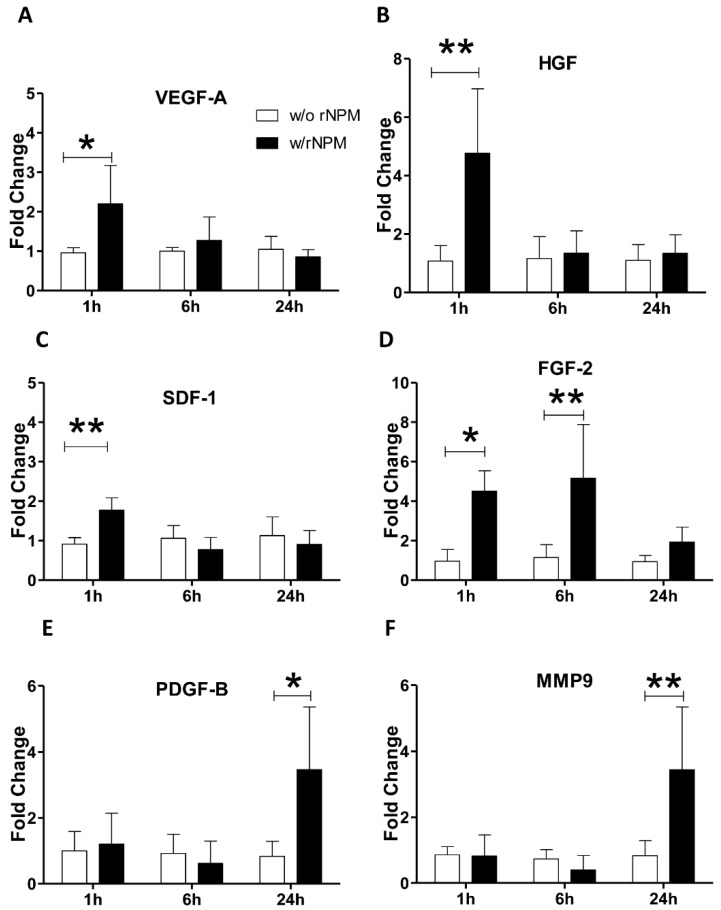
(**A**–**F**). Gene expression regulation of angiogenic genes. (**A**) The graph shows the upregulation of mRNA levels of: (**A**) *VEGF-A*, (**B**) *HGF*, (**C**) *SDF-1*, (**D**) *FGF-2*, (**E**) *PDGF-B*, (**F**) *MMP9* after 1, 6, or 24 h of stimulation with rNPM (200 ng/mL, w/rNPM) compared to the untreated control (w/o rNPM). Data are the results of 3 independent experiments (technical triplicates). * *p* < 0.05, ** *p* < 0.01).

Finally, to further corroborate the angiogenic role of NPM on endothelial cells, we verified whether the exogenous stimulation with rNPM was able to induce the expression of an angiogenic pattern in HUVEC. We found that rNPM enhanced the early upregulation (after 1 h) of angiogenic master genes such as *VEGF-A*, *HGF*, and *SDF-1* (Figure 5A–C, *p* < 0.01 *VEGF-A*, *p* < 0.01 both *HGF* and *SDF-1*). A similar scenario with the significant parallel upregulation of angiogenic genes including *FGF-2, PDFG-B*, and *MMP9* was observed (Figure 5D–F, *p* < 0.05 1 h and *p* < 0.01 6 h *FGF-2*, *p* < 0.05 *PDGF-B*, *p* < 0.01 *MMP9*).

## 3. Discussion

In this study we explored for the first time the extracellular biologic function of NPM in the endothelial system. So far, the contribution of NPM has been largely studied mainly in cancer, overlooking the physiological aspect.

We have confirmed that in a serum withdrawal-based stress approach (in order to mimic growth factors deprivation [18,19]), NPM mediates an early adaptation to acute stress in endothelial cells, highlighting that NPM acts as a key survival mediator in harsh microenvironments. Hallmark of this early stress response is the biological activation of NPM, which compartmentalizes from the nucleus to the cytoplasm, in line with similar studies reporting the strict association between NPM localization and cell response to a wide range of stimuli including UV radiation and hypoxia [20,21].

Importantly, NPM is also released by HUVEC into the extracellular milieu upon stress conditions, and independently from cell necrosis, therefore functioning comparably to well described alarmins such as HMGB-1, IL-33, IL-1, and heat shock proteins, regulating cell migration or invasion [16,22,23]. The role of the NPM as excreted alarmin has been already highlighted, however only after stimulation with pathogen components such as Lipopolysaccharides (LPS) [16].

According to the novel physiological contribution of NPM on endothelial cells, our results highlight that the exogenous NPM exerts a paracrine action in absence of damage on endothelium. Similarly, the exogenous delivery of HMGB-1 has been reported to activate cardiac stem cells, even in the absence of infarction, strengthen the beneficial regenerative contribution of alarmins in physiological conditions [24].

Notably, our data indicate that the stimulation with recombinant NPM fosters the endothelial cellular motility but not cell proliferation, likely due to the arrest in the G0/G1 phase of the cell cycle normally occurring upon serum deprivation in acute conditions [25].

Consistent to an enhanced migration (which represents a major event to initiate angiogenesis) and increased levels of NPM in the extracellular milieu, both the in vitro pro-inflammatory response through the upregulation of ICAM-1 and the in vivo neoangiogenesis are enhanced. The inflammatory/angiogenetic features of NPM have been described mainly in cancer. Accordingly, several studies have reported that activated mouse leukemic monocyte-macrophage cells (RAW 264) are able to release NPM and to consequently induce upregulation of the pro-inflammatory adhesion molecule ICAM-1 [16]. Both ICAM-1 and VCAM-1 are crucial in the trafficking of infiltrated inflammatory cells at the site of injury. However, we found that NPM did not modulate VCAM-1 expression, coherently with an activated status of HUVEC in presence of NPM (VCAM-1 is normally absent on resting endothelial cells), oppositely to ICAM-1, which is constitutively expressed [26,27]. Notably, the exogenous stimulation of endothelial cells with NPM was able to sustain in vivo angiogenesis, where newly formed blood vessels were remodeled and also functional as we found the presence of erythrocytes within [28,29,30].

Finally, we attempted to provide a first molecular explanation of the angiogenic ability of NPM as alarmin to rapidly control main angiogenic genes including *VEGF-A*, *SDF-1*, PDFG-B, *FGF-2*, *HGF*, and *MMP9* (important for stromal remodeling and invasion during the angiogenic process [31]). *VEGF* is the master gene of angiogenesis in vascular cells [32]. So far, most studies have highlighted the role of NPM as the main downstream molecule of *VEGF* only in tumoral angiogenesis or atherosclerosis. Thus, if NPM regulates *VEGF* function in the physiological angiogenesis is still unclear. Serum starvation is known to increase the phosphorylation of NPM in HUVEC. In tumor angiogenesis, NPM acts with different protein partners directly orchestrated by *VEGF*. For instance, the aberrant production of *VEGF* in transformed cells is reported to hyperactivate the Cyclin E/Cdk2 complex, which is responsible for the phosphorylation of NPM at serine-199 and centrosome overduplication because of the shuttling of NPM to the nucleus [33]. Alternatively, both the p19ARF/NPM complex and the nuclear factor-kB (NF-kB) p65 phosphorylation have been found to mediate the translational repression of *VEGF* [34], resulting in the increase of inflammatory genes [3]. Overall, these studies suggest that NPM activates angiogenesis through multiple pathways and molecular partners. In addition, it is important to point out that we explored the angiogeneic role of the recombinant NPM, that usually does not display post-translational modifications [35].

This study has some limitations. We have not investigated if receptors or specific signaling pathways are involved when endothelial cells are stimulated with exogenous NPM. Additional potential mechanisms including the protection of microRNAs from degradation [36] have been found when NPM is released in the extracellular space under particular stress conditions [16,36]. In addition, we cannot rule out that the enhancement of the angiogenesis observed is more ascribable to a reduction of apoptotic mechanisms, DNA damage repair or metabolic process upon stress conditions rather than a true increase of angiogenesis.

Despite this, our data indicate that NPM acts as alarmin with significant paracrine ability and is related to a physiological increase of angiogenesis in endothelial cells. As already described for other alarmins, it is still unclear to what extent NPM might exert protective biological effects on endothelium. Evidence has highlighted the harmful side of inhibiting alarmins, whose presence within the tissue is necessary to trigger significant pro-inflammatory processes.

In particular, chronic conditions seem to cause an imbalanced secretion of alarmins, therefore negatively affecting their regenerative ability [37]. This important issue will also require future investigations.

## 4. Materials and Methods

### 4.1. Cell Culture and Stimulation

Primary Human Umbilical Vein Endothelial Cells (HUVEC) were obtained from donor pool (Cat. N. C2519A, Lonza, Milan, Italy). Cells were cultured in HUVEC complete growth medium (EGM^TM^-2 Endothelial Cell Growth Medium-2 BulletKit^TM^ Lonza, Cat. N. CC-3162. The EGM^TM^-2 complete media is composed by a basal medium EBM^TM^-2 (CC-3156) and SingleQuots^TM^ Supplements (CC-4176) required for the growth of Endothelial Cells) at 37 °C in 5% CO_2_, 95% air, and were used between passage 4 and 6 as previously reported [34,35,36]. When HUVEC were cultured in EGM^TM^-2 complete media, we referred to the serum condition (with Serum) or to the positive control of the experiment, whereas cells treated with EBM^TM^-2 represented the condition without serum (w/o Serum) or the negative control of the experiment. The stimulation with rNPM (126664, Abcam, Cambridge, UK) was performed at a concentration of 200 ng/mL in EBM^TM^-2 medium and therefore in the absence of serum and any growth supplements.

### 4.2. Western Blot

To detect nuclear and cytoplasmic fractions of HUVEC, cells were lysed in a buffer containing 20 mM Hepes (pH 7.4), 10 mM KCl, 2 mM MgCl2, 1 mM EDTA, 1 mM EGTA, 1 mM DTT, and protease and phosphatase inhibitor cocktail (Roche). Once incubated 15 min on ice, cell suspension was passed through a 27-gauge needle 10 times and left for another 20 min of incubation on ice. The lysate was then centrifuged at 700 g for 5 min. Afterwards, the supernatant containing cytoplasmic fraction was collected while the pellet containing nuclei was resuspended with the same buffer and passed through a 25-gauge needle 10 times. Finally, the sample containing resuspended nuclei was centrifuged at 720 g for 10 min and the pellet containing nuclei was resuspended in 2× Laemmli buffer. Proteins were separated by sodium dodecyl sulfate-polyacrylamide gel electrophoresis (SDS-PAGE) and transferred to nitrocellulose membrane by standard procedures. The membranes were blocked with 5% non-fat dry milk powder in 0.05% Tween-20 Tris-buffered saline (TBS-T) for 1 h and immunoblotted with NPM antibody (ab86712, Abcam), Lamin B antibody (sc-6216, Santa Cruz, TX, USA), α-Tubulin monoclonal antibody (DM1A Calbiochem; CP06, Merck, Italy) and in 5% non-fat dry milk powder in 0.05% Tween-20 (TBS-T) overnight at 4 °C. After 4 washes of 15 min each, blots were incubated with TBS-T and incubated with appropriate horseradish peroxidase-coupled secondary antibodies (Amersham, Sigma-Aldrich) and then washed again three times and developed by a chemiluminescence-based detection system (ECL, Amersham, Sigma-Aldrich). Protein amounts were calculated by the Bradford method (Bio-Rad, Hercules, CA, USA). Proteins (15 μg/lane) were resolved by 10% SDS-polyacrylamide gel electrophoresis and transferred to nitrocellulose membrane (Amersham, Sigma-Aldrich). Membranes were probed with specific antibodies for NPM and α-Tubulin as above described.

### 4.3. Immunofluorescence

HUVEC were fixed in PBS with 4% paraformaldehyde and permeabilized in PBS with 0.1% Triton X-100. Coverslips were rinsed and blocked in PBS with 0.2% BSA prior to incubation with antibodies. Fixed cells were incubated with the antibody NPM (ab86712, Abcam) overnight at 4 °C followed by incubation with appropriate secondary antibody coupled to a Fluorocrome (Dako Cytomation) for 1 h at room temperature. The coverslips were washed and stained for the DAPI (Vector Laboratories, Burlingame, USA) to identify nuclei. Images were obtained by ApoTome System (Zeiss) connected with an Axiovert200 inverted microscope (Zeiss); image analysis was then performed with ZEN software (Zeiss).

### 4.4. ELISA and LDH Assay

HUVEC plated in 60 mm dishes (1 × 10^5^ cells/dish) were serum starved overnight. Conditioned medium was collected at 6, 18, and 24 h. NPM levels were measured using an ELISA assay (SEC664Hu, DBA), according to the manufacturer’s instructions. Values were corrected for the protein amounts. LDH was measured using Cytotox96 assay (Promega, Madison, WI, USA) according to manufacturer’s instructions.

### 4.5. Proliferation and Migration

Equal amount of HUVEC (3 × 10^3^/well) were plated in 96-well in triplicate. Cells were serum starved overnight and then treated or not with rNPM at 200 ng/mL (ab126664, Abcam) in EBM^TM^-2. EBM^TM^-2was used as a negative control, whereas full media was used as a positive control in proliferation assay. At the indicated time points, 0, 6, 18, 24, 48, and 96 h, cells were treated with CellTiter 96^®^ AQueous One Solution Cell Proliferation Assay (MTS, Promega, Madison, WI, USA). The rate of proliferation was valuated at 490 nm Absorbance (Magellan™—Data Analysis Software—Life Sciences—Tecan).

Migration was evaluated by Chemotaxis Assays and performed in 48-microwell chemotaxis chambers (Neuroprobe, Cabin John, MD, USA) using 8 μm pore size polycarbonate filters (Costar Scientific, Cambridge, MA, USA) coated with murine collagen type IV (5 μg/mL; Becton-Dickinson, Franklin Lakes, NJ, USA). Briefly, 7 × 10^5^ cells/mL were added to the upper chambers and human recombinant NPM was placed in the lower chambers at the concentrations indicated in the Figure legends. Medium with 0.1% BSA-EBM^TM^-2 and 10% FBS were used as negative and positive controls for migration, respectively. After 4 h incubation at 37 °C in a 5% CO_2_ humidified atmosphere, the chemotaxis assay was stopped. HUVEC on the filter were fixed and stained using Diff Quik Stain kit (Dade AG). Five random fields on the lower face of the filter were counted at 40x magnification and migration index was calculated.

### 4.6. Angiogenesis Assay

Briefly, 200 μL of Matrigel (Corning^®^ Matrigel^®^ Growth Factor Reduced (GFR) Basement Membrane Matrix, Phenol Red-free Cat. N. 356231, Beckton Dickinson, Bedford, MA, USA), was applied to 24-well culture plates. HUVEC (7 × 10^4^ /mL) were seeded on matrigel-coated wells in the presence of EBM^TM^-2 medium supplemented with 200 ng/mL rNPM. Medium with EBM^TM^-2 or 10% FBS were used respectively as negative and positive controls for migration, respectively. The presence of tubular structures was determined 4 h after plating by phase-contrast microscopy. Quantification of tubular structure formation was expressed by the mean number of branching points in five fields by using ImageJ (U.S. National Institutes of Health, Bethesda, ML, USA). Experiments were carried out three times in duplicate.

For in vivo studies, the Matrigel (400 μL, as above-described, Becton and Dickinson) was mixed with rNPM (200 ng/mL) or saline and injected subcutaneously in 5 CD1 male eight weeks mice (Charles River). After 8 days from Matrigel injection, plugs were removed and processed for histological analysis as previously described [24,37]. Briefly, sections (5 µm thickness) were stained with Trichrome-Masson (Bio-Optica) and hematoxylin and eosin staining. The vessels within the plugs were recognized by both morphology and presence of red blood cells. Angiogenesis was evaluated blindly, considering at least three different sections per Matrigel plug; each section was 100 µm from the next. The total number of neovessels (endothelial cells forming a complete lumen with red cells) over the whole area of Matrigel was measured and expressed as number of vessels/mm^2^. All experimental procedures under the protocol SA-IDI-14-MC-2 and complied with the Guidelines of the Italian National Institutes of Health and the Guide for the Care and Use of Laboratory and approved by the Institutional Animal Care and Use Committee.

### 4.7. FACS Analysis

Cells were harvested by trypsinization and low speed centrifugation. The cell pellet was stained for APC anti-human VCAM-1/CD106 or PE anti-human ICAM-1/CD54 in the presence of the dead cell discrimination dye Zombie Acqua (all reagents are from Biolegend). Cell suspensions were washed twice to remove excess reagents and analyzed with a FACSAria I cell sorter (BD Biosciences). Offline analysis of recorded data as flow cytometry standard (FCS) files was performed with FlowJo software (Becton and Dickinson). To minimize artifacts, doublets were excluded by scatter analysis (i.e., events with disproportionate forward-scattered light, FSC-area vs. FSC-height, not shown) and a live cell gate was applied by visualizing cells in an FSC-area vs. Zombie Aqua pseudocolor plot and used as a reference gate to calculate frequencies.

### 4.8. Real-Time PCR

Total RNA was extracted using QIAzol (Qiagen). cDNA was generated by the SuperScript First-Strand Synthesis System (Invitrogen) and real-time PCR was performed with the SYBR-GREEN RT-qPCR method (Qiagen, Germany) using QuantStudio5 Realtime-PCR (10 min at 95 °C, followed by 40 cycles: 95 °C for 15 s, 60 °C for 1 min). mRNA expression was normalized to 18S rRNA. Relative expression was calculated using the comparative Ct method (2–ΔΔCt). The primers used for RT-qPCR are listed in Table 1. All primers are intron spanning except for *FGF-2* and 18S, for which the design of intron spanning primers was not possible. The primers were designed with Primer designer tools (https://www.ncbi.nlm.nih.gov/tools/primer-blast/).

### 4.9. Statistical Analysis

Data collection and statistics data are expressed as mean ± SE. Significant differences between groups of data were assessed using the unpaired Student’s t test or by two-way ANOVA test as reported elsewhere [38]. A *p*-value of *p* < 0.05 was considered statistically significant.

## Figures and Tables

**Table 1:** List of the primers used in the study.

Gene	Primer Sequence
*18S*	Fwd: CATGGCCTCAGTTCCGAAAA Rev: CGAGCCGCCTGGATACC
*VEGF-A*	Fwd: CTACCTCCACCATGCCAAGT Rev: CACACAGGATGGCTTGAAGA
*SDF-1*	Fwd: GCTGGTCCTCGTGCTGAC Rev: GCATGGGCATCTGTAGCTC
*HGF*	Fwd: GGACGCAGCTACAAGGGAAC Rev: CCTTCTTCCCCTCGAGGATT
*FGF-2*	Fwd: GAGACACCCATCCGTGAACC Rev: GGCAGCGTGGTGATGCTC
*PDGF-B*	Fwd: CTCGTCCGTCTGTCTCGATG Rev: GGAAGAAGATGGCGATGGAG
*MMP9*	Fwd: GAACCAATCTCACCGACAGG Rev: GCCACCCGAGTGTAACCATA
